# Knowledge of malaria prevention and control methods and associated factors among rural households in west Belessa district, north west Ethiopia, 2019

**DOI:** 10.1186/s12889-020-09332-x

**Published:** 2020-08-24

**Authors:** Tsigie Baye Aragie

**Affiliations:** University of Gondar, Gondar, Ethiopia

**Keywords:** Malaria, Prevention, Knowledge, West Belessa

## Abstract

**Background:**

In Ethiopia malaria is one of the top ten leading causes of morbidity and mortality. Community knowledge of prevention and control methods had been proven to play an important role in the implementation of effective and sustainable interventions against malaria. This information is limited in the study area. This study aimed to assess knowledge of malaria prevention and control methods and its associated factors among households in West Belessa district, 2019.

**Methods:**

Community-based cross-sectional mixed quantitative and qualitative study was conducted from April to June 2019 in West Belessa district, North West Ethiopia. Multistage sampling was used to select an estimated 770 sample size. A structured questionnaire was used for the quantitative component and semi-structured questionnaire for the qualitative component. Quantitative data were collected by interviewing and qualitative data using focus group discussion (FGD). Quantitative data was coded and entered using Epi info software version 7 then exported to SPSS version 20 for analysis. The binary logistic regression model was fitted to identify the associated factors. Odds Ratio (OR) with 95% Confidence Interval (CI) was used to assess the strength of association. The qualitative data was transcribed manually using the thematic approach.

**Result:**

A total of 770 subjects were included in this study with a 99.5% response rate. Of the total respondents, 75.6% had good knowledge of malaria prevention methods. LLINs and IRS were mentioned by 84.7 and 83.7% respectively. Poorest wealth quintiles [AOR = 0.40, 95% CI: 0.23, 0.73], poor wealth quintiles [AOR = 0.38, 95% CI: 0.21, 0.70], and medium wealth quintiles [AOR = 0.41, 95% CI: 0.22, 0.76], living in Menti Kebele [AOR = 3.07, 95% CI 1.85, 5.08], living in Abay tera kebele [AOR = 2.00, 95% CI 1.21, 3.29] were significantly associated with good knowledge. About all of the focused group discussion (FGD) participants agreed that malaria is a preventable disease and mentioned some of the preventive methods.

**Conclusion:**

Overall there was relatively high knowledge of malaria prevention and control methods. LLINs and IRS were known malaria prevention methods in West Belessa. Wealth index and respondents living kebele are predictors for knowledge of malaria prevention.

## Background

Malaria is one of the leading causes of morbidity and mortality in the world. World health organization (WHO) estimated 228 million cases and 405,000 deaths worldwide in 2018 [1]. In Ethiopia malaria is one of the top ten leading causes of morbidity and mortality [2]. Approximately 60% of Ethiopia’s population lives in malarious areas, and 68% of the country’s landmass is favorable for malaria transmission [3]. Ethiopia has been reported 962,087 malaria confirmed cases and 158 malaria deaths in 2018 [1]. Ethiopia had planned to achieve near-zero malaria deaths and to eliminate malaria in selected low transmission areas by 2020; so as to eliminate malaria in the country by 2030 [4]. To achieve these goals the first option is vector monitoring and controlling through Entomological monitoring and insecticide resistance management [4].

The recommended vector-control operations are effective coverage LLINs and targeted communication about their usage. Regular application of indoor residual spraying with insecticides [5]. Other vector control activities including larval control through environmental management and chemical larvicides are also practiced in areas where such interventions are appropriate and expected to have a significant impact [6].

Knowledge of community contributes immensely to sustainable control of endemic diseases such as malaria [7]. Federal ministry of health (FMOH) of Ethiopia targeted by 2020, all households living in malaria-endemic areas will have knowledge about malaria prevention and control [3]. The Ethiopian national malaria indicator survey report shows that 63 and 77% of the respondents knew LLIN as a malaria prevention tool in 2011 and 2015 respectively [6].

In different literature, there are factors that can affect an individual’s knowledge of malaria prevention and control methods. Among socio-demographic variables individuals’ resident, educational status, and wealth quintile are associated factors with malaria prevention and control knowledge [8–10].

Nowadays, there is no effective vaccine or no effective drug for mass chemoprophylaxis against malaria. Thus, proper know-how of prevention methods is crucial. In Ethiopia, despite the intense activities pertaining to the distribution of LLINs, performing indoor residual spraying, and the provision of anti-malarial drugs free of charge, malaria is still a major community health problem. Many questions about malaria remain unanswered. These include the extent to which people are aware of the benefit of LLINs and IRS the value they give them [11]. So this study was aimed to assess knowledge of malaria prevention and control methods and associated factors among rural households in West Belessa district 2019.

## Methods

### Study design and setting

Community-based cross-sectional mixed quantitative study and qualitative study was conducted from April to June 2019.

This study was conducted in West Belessa district which is situated in Amhara Regional State, North West Ethiopia. Ethiopia is one of the countries in East Africa with 1.104 million km^2^ areas and 112,078,730 population in 2019. It has 9 regional states and 670 rural districts. West Belessa is one of these districts.

West Belessa is, situated at an altitude of 1501 to 3000 m above sea level. The capital city of West Belessa is Arbaya. It is located at a latitude and longitude of 12^o^ 15′N and 37° 45′E respectively. High Rainfall is registered from June to August with a shortage and heavy rain intermittently. The mean monthly temperature is 33.5^0^c. There are mountainous, lowlands, and water bodies including a known river Mena. The district has 3 climatic zones, which are hot zones (kola) 60%, cold zones (dega) 35%, and moderate zones (weyna dega) 5%. From the total district land area 92.9% are malarious. In 2019 the estimated total population of the district was 197,326.

### Study population and sampling technique

The source population for this study was all households in 23 malarious rural kebeles. There are a total of 25 kebeles in the district. The study population was all households in 5 selected malarious kebeles. The sample size for the survey was determined by using a single population formula. We used the proportion of knowledge towards malaria prevention methods (64.8%) [12], 95% CI, and a margin of error of 0.05 then 350 has been calculated. By adding 10% non-response rate and design effect 2 the final sample size became 770. There are 25 total kebeles in West Belessa. First of all these kebeles divided into 3 clusters (Malarious, non-malarious and urban). From the rural malarious kebeles’ 5 kebeles were selected by using the lottery method. The sample size was allocated proportionally based on Keble’s population. Using household registration from health post, we selected households by systematic sampling every 15 households. The interviewees are household heads.

### Inclusion and exclusion criteria

#### Inclusion criteria

All households’ head in the selected kebeles and have been living there for at least 6 months before the interview.

#### Exclusion criteria

Very sick individuals who were unable to communicate excluded from the study.

### Data collection methods and data quality control

The data collection tool was adopted from 2004 WHO/UNICEF guidelines for core population coverage survey [13] with some modifications. Originally prepared in English then translated into the local language (Amharic) then back to English to ensure reliable information. Twelve numerators who had graduated from college were recruited and trained about the purpose of the study; how to approach respondents; how to obtain written consent and overview of malaria prevention methods for 1 day. An interviewer-administered questionnaire was used. Completeness of questionnaire was checked every day and incomplete questionnaires were returned to the data collectors on the following day for correction by re-visiting the households. Absence households were revisited on the following days. Pre-testing was conducted in one of the malarious kebeles out of the study area.

A total of 4 FGDs were conducted in 4 kebeles (Abay tera, Kalay, Aswagari, and Menti kebeles). Participants with full of information about malaria prevention and control methods were selected for the discussion by using health extension workers as a key informant in each kebele. The FGD had 6 to 10 participants in each group and comprised a total of 30 study participants in 4 groups. Each FGD was conducted by recording sounds and taking notes. The selected interviewees were expected to answers the semi-structured questionnaires. The content of semi-structured questionnaires were issues about knowledge of malaria prevention and control methods.

### Variables of the study

Knowledge about malaria prevention and control was a dependent variable of the study. Knowledge: A study participant who scored above or equal to the median score of knowledge questions was considered as having good knowledge and others considered as having poor knowledge [14]. The media was 7 out of 12 malaria prevention knowledge questions.

Age; sex, marital status, occupation, religion, ethnicity, address, family size, educational status, and wealth index are independent variables.

Wealth quintiles: determined using durable household assets. A total of 18 different durable assets were identified and assigned as dummy variables. After adjusting and coding we used multivariate analysis i.e. principal component analysis after we re-categorized into five different wealth quintiles, each with an approximately equal number of households.

## Data processing and analysis

Quantitative data entered and coded using Epi info version 7 and exported to SPSS version20. Variables were analyzed using logistic regression in SPSS to determine the association between the variables determined by looking at the level of significance of 0.05 with a 95% Confidence Interval (CI).

Qualitative data were transcribed in the original language of interview first word by word from the audiotapes and field notes, then it was translated to English for analysis. The primary theme was produced through manual coding using a pen of different colors, then it was pooled into broader concepts to form main themes.

### Ethical consideration

Approval to carry out the study was sought and obtained from the University of Gondar Ethics Review Board. Written consent was obtained from all study participants after a detailed explanation of the purpose of the study.

## Result

### Socio-demographic characteristics

A total of 766 households have participated in this study with a response rate of 99.5%. Out of a total participant, 419 (54.7%) were male. The mean age of the respondents was 40.6 (SD = ± 12.23) years. Almost all of the respondents 760 (99.2%) were orthodox Christian, and the majority of the respondents 691(90.2%) were married. Concerning the educational status majority, 672 (87.7%) of the participants from the surveyed households were illiterate. About half 373 (48.7%) of the respondents living houses had 2 sleeping places and the mean sleeping place per household was 1.64 (SD ± 0.75). The economic status of the respondents was assessed in wealth index showed, 18.8, 20.5, 18.9, 19.1, and 22.7% of the respondents ranked as wealthiest, wealthy, medium, poor, and poorest respectively. (Table 1).

**Table 1:** socio-demographic characteristics of respondents for knowledge towards malaria prevention and control methods in West Belessa, North west Ethiopia, 2019

Variable	*N* = 766	Frequency	Percent
Age	18–24	50	6.5
	25–34	199	26
	35–44	242	31.6
	45–54	173	22.6
	55–64	70	9.1
	> 64	32	4.2
Sex	Male	419	54.7
	Female	347	45.3
Religion	Orthodox	760	99.2
	Others	6	0.8
Educational status	Illiterate	672	87.7
	Literate	49	6.4
	Formal education	45	5.9
Marital status	Married	691	90.2
	Divorced	26	3.4
	Not married	36	4.7
	Widowed	13	1.7
Number of sleeping spaces	1	373	48.7
	2	318	41.5
	≥3	75	9.8
Wealth index	Poorest	174	22.7
	Poor	146	19.1
	Medium	145	18.9
	Wealthy	157	20.5
	Wealthiest	144	18.8

### Knowledge of malaria prevention and control

**Fig. 1:**
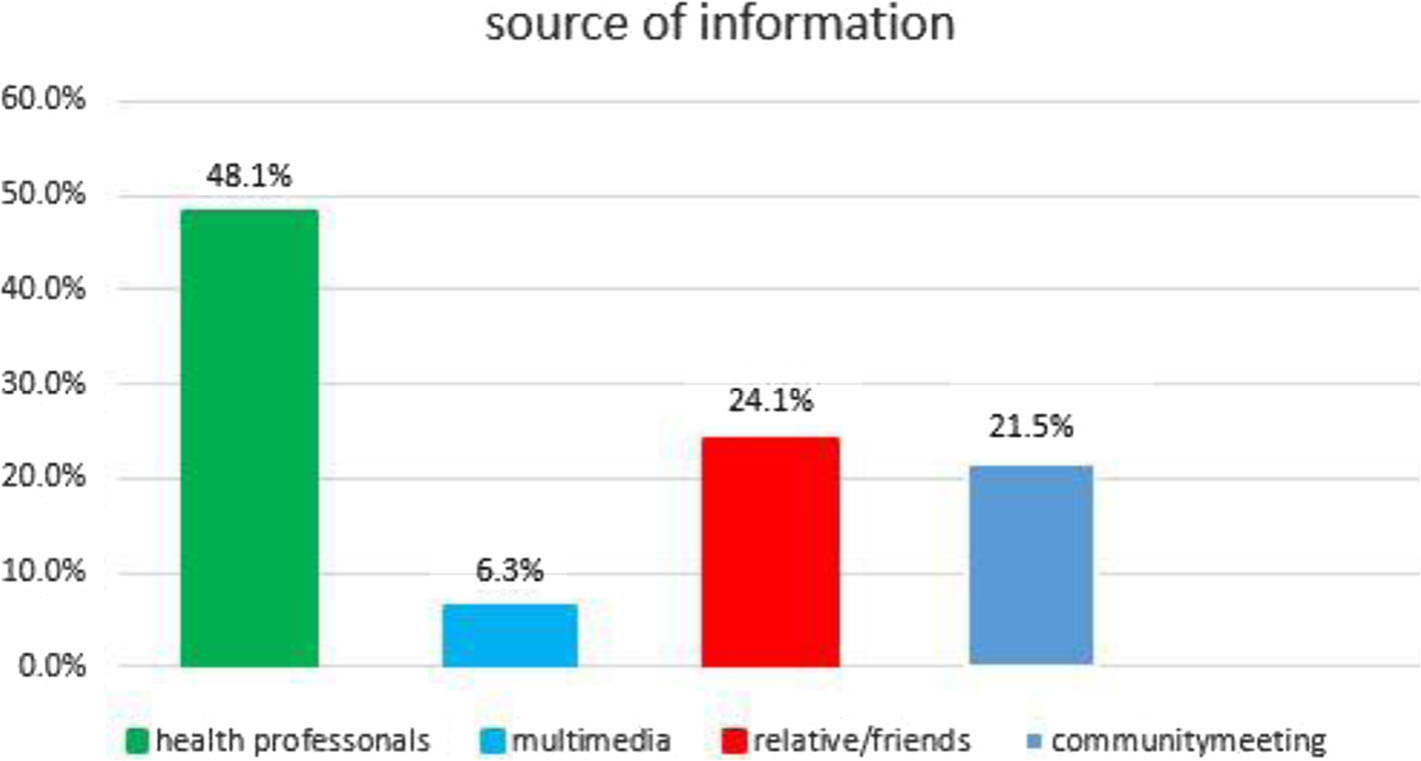
Source of information about malaria prevention methods in West Belesa, North West Ethiopia, 2019

In this study majority (93.2%) of the study participants, heard information about malaria. Of those about half, 48.1% got the information from health professionals. (Fig. 1).

According to the FGD malaria was known as Weba or Nidad in West Belessa interchangeably. The majority of the respondents (94.6%) believed that malaria is a preventable disease. Regarding the specific type of malaria preventive methods most frequently mentioned prevention methods were LLINs (84.7%) and IRS (83.7%) but the least frequently mentioned prevention methods were other malaria prevention methods (7.8%). With regard to the knowledge of mosquito breeding sites, most (87.8%) of the respondents mentioned stagnant water as a breeding site for malaria vector (mosquito). About half (45.9%) of the respondents mentioned that at day time mosquito rests in unclean vegetation. (Table 2).

**Table 2:** Malaria prevention knowledge of study participants in West Belessa, North west Ethiopia, 2019

Variable	Yes (%)	No (%)
Preventability of malaria	725(94.6)	41(5.4)
Methods of malaria prevention		
IRS	641(83.7)	125(16.3)
Using LLIN	649(84.7)	117(15.3)
Source reduction	526(68.7)	240(31.3)
Using drug prophylaxis	350(45.7)	416(54.3)
Clear the vegetation	299(39.0)	467(61.0)
Others prevention methods	60(7.8)	706(92.8)
Breeding site for malaria vector mosquitoes		
Stagnant water and swampy areas	673(87.9)	93(12.1)
Waste material	98(12.8)	668(87.2)
Running water	46(6.0)	720(94.0)
Malaria vector mosquitoes resting during day time		
Unclean vegetation	349(45.6)	417(54.4)
In the house	225(29.4)	541(70.6)

Other malaria prevention methods are close door/window, window screening, and aerosol spray

Overall; the total participants 579(75.6%) (With 95% CI, (72.6, 78.6)) had good knowledge about malaria prevention and control measures, The median knowledge score for all respondents was 7 out of a possible 12 points (IQR = 7–8).

In the FGD; about all of the participants in the discussion agreed that malaria is a preventable disease. Majority of the discussants believed that Environmental management such as compact, drainage, and clearing the vegetation as malaria prevention methods. However, some of the participants also mentioned hygiene conditions (self-hygiene, using toilet, and eating fresh food) as malaria prevention methods. A 45 years old man in Kalay Kebele FGD quoted that “using toilet and hygienic condition of the environment prevents all disease including malaria”. Most of the people in the discussion mentioned that the insecticide bed net as a major malaria prevention method and only a few of them mentioned IRS as a malaria prevention method. One of the discussants from Menti kebele FGD mentioned: “unlike the previous times, nowadays we are preventing malaria by bed net and anti-malaria spry.” In another FGD a female participant in Aswagari “We can prevent malaria through cleaning our environment and using the bed net.”

Some participants explained that the IRS can poison the food if there is carelessness and can kill useful insects like honey bees. For example, a male participant in Menti kebele explained: “It is real when the bee rests on the wall the chemical can kill the bee.” Other malaria prevention methods are not mentioned by the discussant. Most of the discussants mentioned that both LLIN and IRS decreased their effect as compared to the previous brands.

### Determinants of knowledge towards malaria prevention and control

From the multivariable logistic regression model, the odds of having good knowledge is decreased by 60% among respondents in a poorest wealth quintile as compared to the wealthiest with [AOR = 0.40, 95% CI: 0.22, 0.73]. Similarly, the odds of having good knowledge is decreased by 62% among respondents in the poor wealth quintile as compared to the wealthiest [AOR = 0.38, 95% CI: 0.21, 0.70]. Moreover, the odds of good knowledge is decreased by 59% among respondents in the medium wealth quintile as compared to the wealthiest [AOR = 0.41, 95% CI: 0.22, 0.76]. Respondents who live in Abay tera kebele and Menti kebele had 2 and 3 times increased odds having a good knowledge than Aswagari kebele with [AOR = 2.00, 95% CI 1.21, 3.29], and [AOR = 3.07, 95% CI 1.85, 5.09] respectively. (Table 3).

**Table 3:** determinants of malaria prevention and control knowledge in West Belessa, North West Ethiopia, 2019

Variable	Response	Knowledge		COR(95%CI)	AOR(95%CI)	*P*-value
		Good (%)	Poor (%)			
Resident’s Kebele	Kalay	85(11.1)	29(3.8)	1.48(0.88–2.48)	1.50(0.88–2.56)	0.14
	Abaytera	125(16.3)	33(4.3)	1.91(1.17–3.11)	2.00(1.21–3.29)	0.007
	Menti	189(24.7)	29(3.8)	3.29(2.00–5.39)	3.07(1.85–5.08)	< 0.001
	Amstya	55(7.2)	33(4.3)	0.84(0.50–1.42)	0.82(0.48–1.41)	0.48
	Aswagari	125(16.3)	63(8.2)	1	1	
Sex	Female	246(32.1)	101(13.2)	0.63(0.45–0.88)	0.71(0.50–1.01)	0.058
	Male	333(43.5)	86(11.2)	1	1	
Educational status	Illiterate	504(65.8)	168(21.9)	1.09(0.55–2.16)	1.09(0.53–2.23)	0.82
	Literate	42(5.5)	7(0.9)	2.18(0.77–6.16)	1.84(0.62–5.41)	0.27
	Formal education	33(1.6)	12(4.3)	1	1	
Wealth index	Poorest	121(15.8)	53(6.9)	0.37(0.21–0.65)	0.40(0.22–0.73)	0.003
	Poor	104(13.6)	42(5.5)	0.40(0.22–0.72)	0.38(0.21–0.70)	0.002
	Medium	105(13.7)	40(5.2)	0.42(0.23–0.77)	0.41(0.22–0.76)	0.005
	Wealthy	125(16.3)	32(4.2)	0.63(0.34–1.16)	0.66(0.35–1.23)	0.19
	Wealthiest	124(16.2)	20(2.6)	1	1	

## Discussions

This study mainly investigated the knowledge of malaria prevention and control methods and associated factors among rural household malarious areas of West Belessa.

In this study above three fourth (75.6%) of the respondents had compressive good malaria prevention knowledge which is higher than a study done in Amhara region (64.8%) and Areka (50.4%) [12, 14]. This could be due to the study season difference because the compared studies were conducted in none malarious seasons whereas this study has been conducted in the malaria transmission season. In malarious seasons promotion of malaria prevention is conducted which could increase their awareness.

In a specific manner in this study, LLIN and IRS are well known where 84.3 and 83.7% of the respondents explained LLIN and IRS as prevention methods of malaria respectively. This is higher compared with studies done in Tanzania, Gini, Assosa, and Zones of Amhara region [8, 12, 15, 16]. similarly, environmental managements like breeding site reduction and clearing was mentioned by 76.1% of respondents as malaria prevention method which is higher than studies done in Arba Minch and Gurage [17, 18]. Despite this closing windows and doors at an early time in the evening and using aerosol is mentioned at a lower rate. Only 3.7 and 2.3% of the respondents mentioned these as malaria prevention methods respectively. This is lower compared to a study done in Arba Minch [17]. The high knowledge level of IRS, LLIN, and environment management could be attributed to the fact that these were frequently promoted and used by the community as the first options of malaria prevention methods in the district and low level of knowledge of close door/window, using aerosol, and window screening could be attributed to the fact that these were not being promoted as a malaria control methods in the study area as confirmed in the FGD because none of the participants mentioned these as malaria prevention options.

In this survey poorest, poor, and Medium wealth quintile respondents have about 60, 62, and 59% less good knowledge respectively as compared with the wealthiest wealth quintiles. This is in line with studies done in Malawi and Assosa [19, 20]. Which could be due to the fact that individuals from high wealth index households have better information on access since they can buy a radio and other information sources.

People who live in Menti kebele and Abay tera kebele have about 3 and 2 times good knowledge as compared with people who live in Aswagari kebele, the evidence could show the strength in health extension program and health education [21]. for example in Menti kebele the health extension program was headed by three health extension workers as compared with other kebeles which was headed by one and two health extension workers.

As limitation this study was carried out in rural household of west Belessa. It is not good representative of the urban community. Further, this analysis was unable to establish causality due to cross sectional nature of the design and use of logistic regression.

## Conclusions

In West Belessa. Even though there were good LLIN, IRS, and environmental management knowledge. Other malaria prevention measures such as screening and closing at early time windows and doors, use of aerosol spray, etc. are not well known. Wealth index and respondents living kebele are predictors for malaria prevention knowledge of malaria. Programmers, partners, and implementers should prioritize low socio economic groups in the health education. Similarly, they should give health education regarding the use of easily accessible personal protection methods such as screening windows and doors as well as other malaria prevention methods such as using an aerosol spray. Further research is needed for the efficacy of IRS and LLIN. Low socio-economic groups, low socioeconomic status respondents need attention in health education and promotion.

## Supplementary information

**Additional file 1.** Data collection tool for Knowledge of malaria prevention and control methods.

## Data Availability

The data could be accessed for every one based on requests.

## Abbreviations

AOR: Adjusted odds ratio
CI: Confidence interval
FGD: Focus group discussions
FMOH: Federal Ministry of Health
IQR: Inter quartile range
IRS: Indoor residual spray
LLIN: Long lasting insecticidal nets
OR: Odds ratio
SD: Standard deviation
UNICEF: United Nations Children’s Fund
WHO: World Health Organization
