# Supplementary material for: Knowledge of malaria prevention and control methods and associated factors among rural households in west Belessa district, north west Ethiopia, 2019
Source: BMC Public Health. 2020 Aug 24;20:1275. doi: 10.1186/s12889-020-09332-x (PMC7445930; doi:10.1186/s12889-020-09332-x)
Supplement: Supplementary file 1 — Additional file 1. Data collection tool for Knowledge of malaria prevention and control methods. [file 12889_2020_9332_MOESM1_ESM.docx]

**Additional file 1**

**Data collection tool for Knowledge of malaria prevention and control methods.**

|  | 1. **Socio-Demographics** |  |
| --- | --- | --- |
| S.noo | Questions | Alternative answers |
| 001 | Kebele | ________________ |
| 002 | How many family members are there in the house? | __________________ |
| 003 | How many living rooms does your house have? | _________________ |
| 004 | What is the sex of respondents? | 1. Male 2. Female |
| 005 | How old are you? | ____________________years |
| 006 | What is your marital status? | 1. Married 2. Divorced 3. Not married 4. Widowed |
| 007 | What is your occupation? | 1. No work 2. Government employee 3. Merchant 4. Daily laborer 5. Farmer 6. house wife 7. 88.Others (specify)--------------------- |
| 008 | What is your education status? | 1. Illiterate 2. Literate 3. Primary completed grade(1 – 8) 4. Secondary completed grade(9-12) 5. Diploma(10+3) 6. Degree & above |
| 009 | What is your religion? | 1. Orthodox 2. Muslim 3. Protestant 4. Catholic 5. 88.Others (specify)-------------- |
| 010 | Ethnicity | 1. Amhara 2. Oromo 3. Tigre 4. Qimant 5. Others specify------------------------- |
| 011 | Does this household own any livestock, herds, other farm animals, or poultry? | 1. Yes 2. No |
| 012 | If the answer is yes for question no. 011 list type and number of animals. | Number   1. Cows/Bulls _________ 2. Other cattle ________ 3. Horse/Donkey ________ 4. Goats/Sheep ________ 5. Chickens _________ 6. Others/specify___________ |
| 013 | Does any member of this household own any agricultural land? | 1. Yes 2. No |
| 015 | Which materials do you have in the house? | 1. Radio 2. Mobile telephone 3. Table/chair 4. Bed with cotton / sponge/ mattress. 5. Kerosene lamp |
| 016 | Does any member of this household own the following: | 1. An animal-drawn cart? 2. A watch? 3. A mobile phone? 4. A Bajaj? 5. A motorcycle or motor scooter? 6. A bicycle? |
| 017 | What is the main source of drinking water for members of your household? | 1. piped water 2. dug well 3. water from spring 4. rainwater 5. surface water (river/dam) 6. other/specify |
| 018 | What type of fuel does your household mainly use for cooking? | 1. Kerosene  2. Charcoal 3.Wood  4.Straw/shrubs/grass  5.Agricultural crop  6.Animal dung.  88. Other/specify _____________________ |
| 019 | Is the cooking has a separate room? | 1. yes  2. no |
| 020 | Is there latrine which the household uses? | 1. Yes 2. no |

| **2.Knowledge** | | | |
| --- | --- | --- | --- |
| **010** | Can we prevent malaria infection? | 1. Yes 2. No |  |
| **011** | How can we prevent malaria infection/transmission? | 1. IRS  2. Source reduction/Environmental management  3.Drugs (prophylaxis)  4.ITNs utilization  5. Sleeping with fully dressed  6. Closing doors and windows in a time  7. If other, specify ________________ |  |
| **013** | Have you ever heard about mosquito net (if no, go to Q7)? | 1.yes  2.No |  |
| **014** | What is the purpose of using mosquito net? | 1.Protect from mosquito bite  2.Protect from other insects  3.Sleep better  4.To get warm  88.Others/Specify -------- |  |
| **015** | What are the purpose of Insecticide residual spraying in your house? | Yes No  1.To kill mosquitoes 1 2  2.To kill other insects 1 2  3.To prevent malaria 1 2  88.Others/Specify ------------ |  |

1. **Focus Group Discussion guide For qualitative**
2. **Identify the Knowledge regarding perception of community regarding malaria prevention methods.**

**A. Can you tell us the perceptions in relation to malaria Prevention methods in your community? Probe for:**

1. Any traditional/cultural methods for prevention for malaria
2. Any modern methods for prevention for malaria
3. What does the community make sure that their children or pregnant women in their household won’t get malaria?
4. **Identify key behavioral perceptions use of** LLINs
5. **How do you see the perception of the community to wards LLINs? Probe:**
6. What is the purpose of using LLINs?
7. Have you heard of any health and environmental hazards which are believed to be caused by LLINs? Probe for these side effects
8. **Understand the perceptions of the community about IRS**

**A. How do you see the perception of the community to wards IRS? Probe:**

1. What is the purpose of using IRS?
2. Have you heard of any health and environmental hazards which are believed to be caused by IRS? Probe for these side effects
